# Case report: complete response and long-term survival on third-line immunotherapy in patient with pleural mesothelioma

**DOI:** 10.3389/fonc.2024.1388829

**Published:** 2024-08-29

**Authors:** Željka Jurić Čuljak, Snježana Tomić, Kristina Šitum, Tihana Boraska Jelavić

**Affiliations:** ^1^ Department of Pulmonary Diseases, University Hospital of Split, Split, Croatia; ^2^ Department of Pathology, Forensic Medicine and Cytology, University Hospital of Split, School of Medicine, University of Split, Split, Croatia; ^3^ Department of Radiology, University Hospital of Split, Split, Croatia; ^4^ Department of Oncology, General Hospital Dubrovnik, Dubrovnik, Croatia; ^5^ University Department for Health Studies, University of Split, Split, Croatia

**Keywords:** pleural mesothelioma, bevacizumab, immunotherapy, pembrolizumab, chemotherapy, case report

## Abstract

**Introduction:**

Pleural mesothelioma (PM) is a rare neoplasm with median survival time range from 8 to 14 months from diagnosis, and the 5-year survival rate less than 10%, indicating a poor prognosis. The standard treatment for unresectable PM for a long time has been polychemotherapy with pemetrexed and cisplatin for fit patients. Currently, the combination of the anti PD-1 inhibitor nivolumab and the anti-CTLA4 inhibitor ipilimumab has been recognized as the best possible frontline therapy (especially in the sarcomatoid or biphasic type) due to improved outcomes compared to the standard chemotherapy combination. There are still no established predictive biomarkers for any type of systemic therapy in this disease.

**Case presentation:**

Patient who presented with cough and dyspnea has been diagnosed with advanced epithelioid type PM in May 2016. He was treated with three lines of therapy, including an antiangiogenic agent and immunotherapy with pembrolizumab in the third line. Immunotherapy with the PD-1 inhibitor pembrolizumab achieved a complete and prolonged response that transferred to long- term survival. Seven years from diagnosis, the patient is still alive. Histological findings showed an unusually immune-inflamed tumor microenvironment possibly leading to excellent response on immunotherapy.

**Conclusions:**

The course of the disease in our patient points out that we need better predictive biomarkers to direct the treatment algorithm, as some of the patients, although chemorefractory to the best chemotherapy option, can sustain great benefit of second-line chemotherapy in combination with antiangiogenic agent, and especially immunotherapy, even in late lines of therapy.

## Introduction

Mesothelioma is a rare neoplasm that most commonly arises on the mesothelial surfaces of the pleural cavity (1). The epithelioid subtype is the most common pleural mesothelioma (PM) subtype, followed by the sarcomatoid and biphasic subtype, respectively (2). PM is an aggressive inflammatory cancer associated with exposure to asbestos (3). Its incidence has continued to increase worldwide, and it is not expected to drop until sometime between 2015 and 2030 (4). The median survival time ranges from 8 to 14 months from diagnosis, and the 5-year survival rate is less than 10%, indicating a poor prognosis (5).

Standard treatment for unresectable PM for a long time has been polychemotherapy with pemetrexed and cisplatin for fit patients (6). In a very limited population of highly selected patients, surgery and radiation therapy can be performed in the trimodal treatment strategy (6, 7). However, nearly all patients with PM progress during or after first-line treatment. Since immune checkpoint inhibitors (ICI) have revolutionized the therapeutic options and outcomes of advanced non-small cell lung cancer (NSCLC), several clinical trials have also been launched in PM. Currently, the combination of the programmed cell death protein 1 (PD-1) inhibitor nivolumab and the cytotoxic T- lymphocyte associated protein 4 (CTLA-4) inhibitor ipilimumab has been recognized as the best front-line therapy due to improved results compared to the standard chemotherapy combination (8). Most recently, first-line chemotherapy with cisplatin pemetrexed combined with the checkpoint inhibitor pembrolizumab has been shown to prolong overall survival in comparison to the same chemotherapy alone (9).

An antiangiogenic monoclonal antibody bevacizumab administered concurrently with a combination of cisplatin-pemetrexed and as a maintenance has resulted in prolonged overall survival compared to chemotherapy alone in first-line treatment (10). Bevacizumab and other antiangiogenic drugs have been investigated in further lines of treatment as well, with mixed results (11). Recently, the results of a phase II randomized study (RAMES) on second –line combination of gemcitabine and antiangiogenic drug ramucirumab have been published. This combination prolonged overall survival in comparison to monochemotherapy with gemcitabine, postulating it as a new possible standard of care in this line of treatment (12). Next lines of therapies are palliative in nature and usually consist of sequential use of monochemotherapy (gemcitabine, vinorelbine).

Therefore, we present a case of a patient diagnosed with advanced PM who was treated with three lines of therapy, including antiangiogenic agent in the second line and immunotherapy with pembrolizumab in the third line. Seven years from diagnosis, the patient is still alive and in complete remission.

## Case presentation

### Clinical history

A 67-year-old man, with a personal history of arterial hypertension and anxiety-depressive disorder, presented in April 2016. with cough and dyspnea. Diagnostic workout (chest radiograph, CT scan of the thorax, and bronchoscopy) has raised suspicion of pleural mesothelioma. Fluorodeoxyglucose (18F-FDG) positron emission computer tomography (PET-CT) was performed in the same month that showed metabolically active right pleural plaques, with possible involvement of mediastinal lymph nodes (right paratracheal and phrenicocardiac region). In May 2016. the patient was operated by the sole decision of the thoracic surgeon. Right extrapleural pneumonectomy and mediastinal lymphadenectomy was performed. The pathohistological finding confirmed pleural mesothelioma (diffuse epithelioid type) with mediastinal lymph nodes involved, pathological stage pT3N2 according to the AJCC, seventh edition staging system.

Postoperative PET-CT, three months after surgery, described intense focal metabolic accumulation of fluorinated glucose (SUVmax = 6.5) in the caudal part of the right costal arch, as well as along the gastric cardia (SUVmax =8.9).

The patient was transferred to the University Hospital for further treatment. After multidisciplinary discussion, the combination of cisplatin and pemetrexed in standard doses as first-line chemotherapy was administered every 21 days for 2 cycles. Morphological evaluation was performed after 2 cycles of chemotherapy by CT scan of the thorax and upper abdomen. According to the response evaluation made by radiologist, the patient had progression of the disease. The enlargement of lesions in the right thoracic wall with infiltration into the muscular layer along the scapular and subscapular region was described, as well as thickening of the infiltrative layer on the side of the removed parietal pleura, while the nodule located in the gastric cardia region was incorporated into the infiltrative consolidation greater than 4 cm in diameter with a tendency to narrow the esophageal lumen.

Due to the fact that the patient was still in good general condition, without toxicity from previous chemotherapy, the multidisciplinary team (MDT) proposed second line treatment with gemcitabine. Discussion with the patient about the expected benefit of monogemcitabine chemotherapy led to a shared decision to incorporate bevacizumab alongside it. The patient was financing bevacizumab (off-label) by himself. From October 2016. to December 2017., the patient received 15 cycles of this combinational therapy (gemcitabine 1000 mg/m^2^ day 1 and 8, and bevacizumab 7.5 mg/kg day 1 of every 3-weeks cycle) with the best radiological response being partial response (PR), and the largest reduction in size was in a lesion in the gastric cardia area.

**Figure 1:**
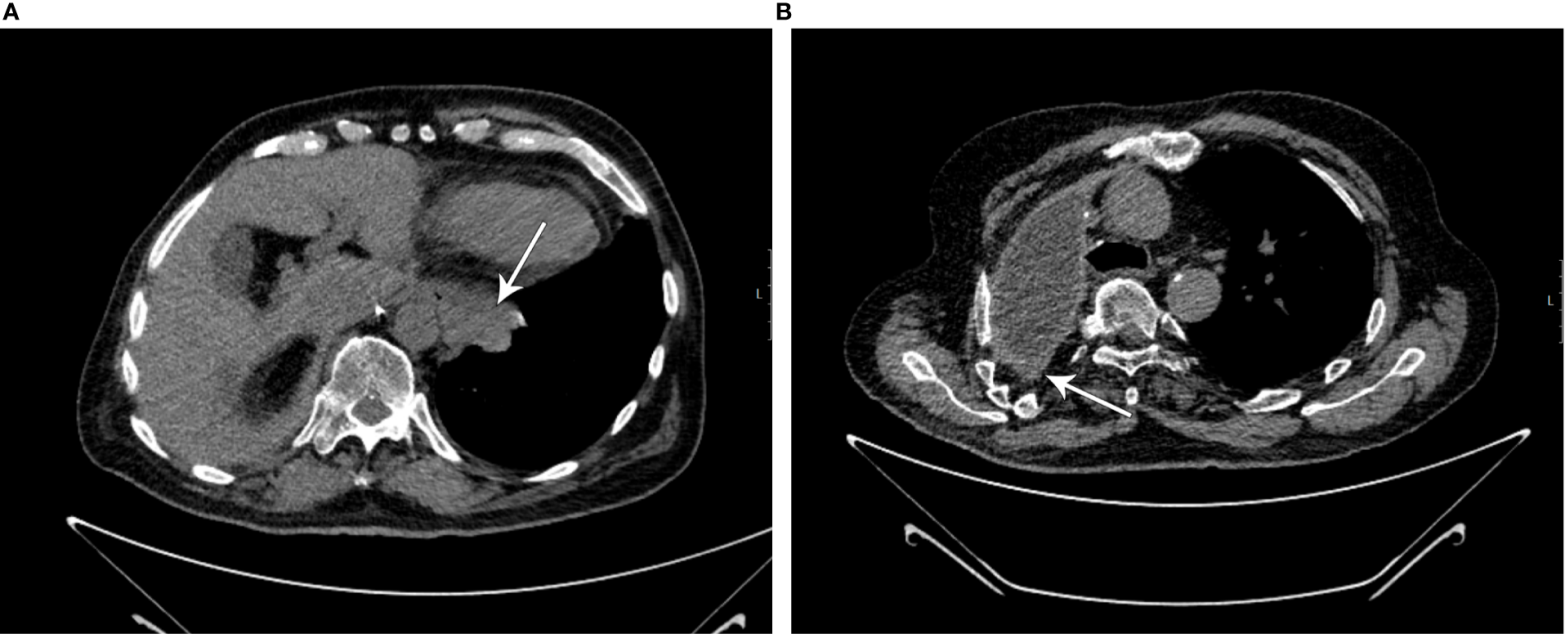
**(A, B)** CT scans showing progression on second-line therapy (marked by white arrows; initial scans for pembrolizumab treatment).

**Figure 2:**
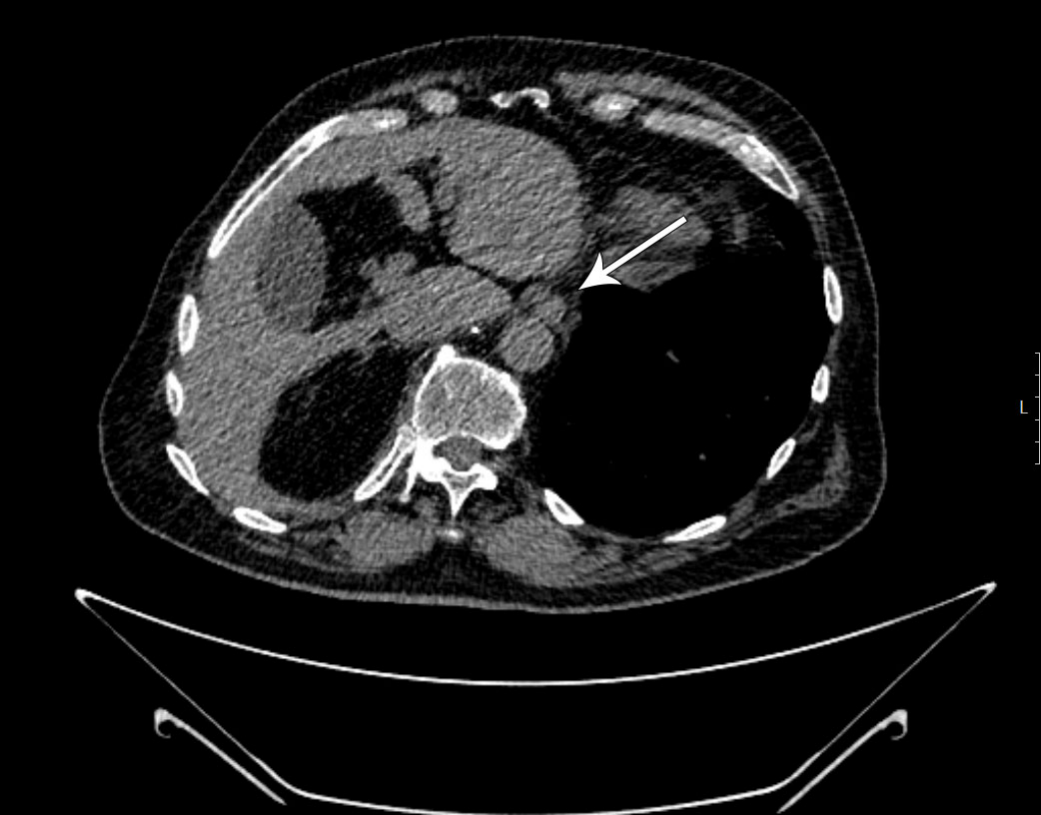
Complete regression of the tumor infiltrate along the distal esophagus and cardia (marked by white arrow).

**Figure 3:**
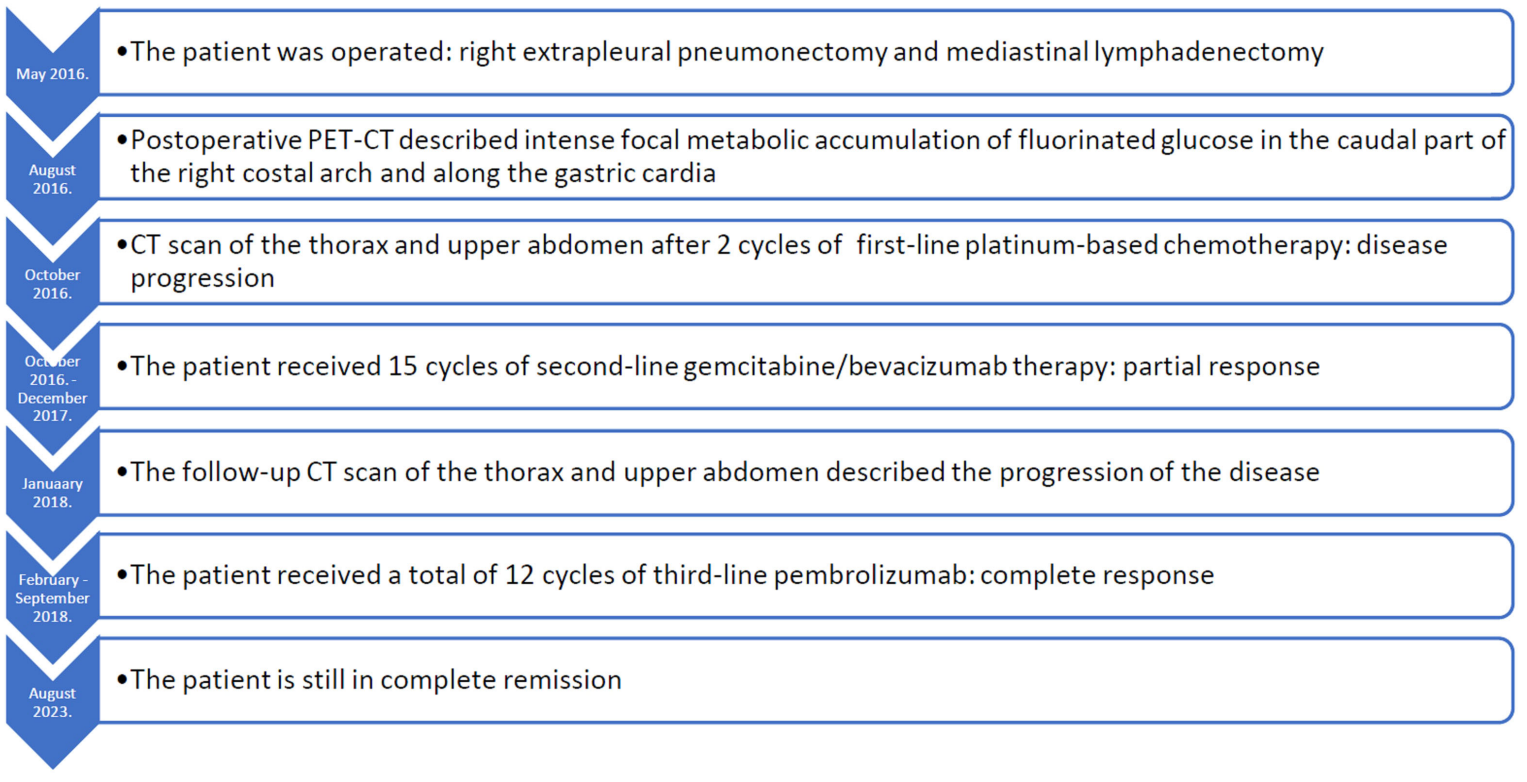
Timeline of this patients course of the disease.

The follow-up CT scan of the thorax and upper abdomen in January 2018. described the progression of the disease with tumor involvement of the contralateral (left) pleura, as well as occurence of new enlarged mediastinal lymph nodes, and the recurrence of the node in the gastric cardia region (**Figures 1A, B**). The patient was offered third-line vinorelbine monochemotherapy, which he refused. At that time, the first reports on immunotherapy in PM were published, and after a thorough discussion with the patient, immunotherapy treatment as an option was proposed. Additional pathohistological analysis revealed positive PD-L1 staining in 2% of tumor cells in the primary tissue specimen, and we have chosen pembrolizumab as a next-line treatment. The patient was financing the treatment by himself (drug was given off-label). From February to September 2018. the patient received a total of 12 cycles of pembrolizumab, administered every 3 weeks. The radiological evaluation showed a complete response to therapy (**Figure 2**). Treatment was stopped in November 2018., due to the development of grade 3 pneumonitis for which the patient was hospitalized and treated with high doses of oral corticosteroids, intravenous antibiotics, oxygen and other supportive measures. Patient recovered completely from pneumonitis. Given the complete response and significant pulmonary toxicity of the treatment and acknowledging that the patient had right pneumenectomy, the MDT decided to stop further treatment with pembrolizumab and carefully monitor the patient. The clinical and radiological evaluation was continued every 3 to 4 months, and after two years of follow-up every six months. The last follow-up examination was in August 2023., and the patient is still in complete remission, with no sequelae to treatment and living with a very good quality of life. Timeline of this patients disease course is shown in **Figure 3**.

### Histological findings and molecular analysis (next generation sequencing)

**Figure 4:**
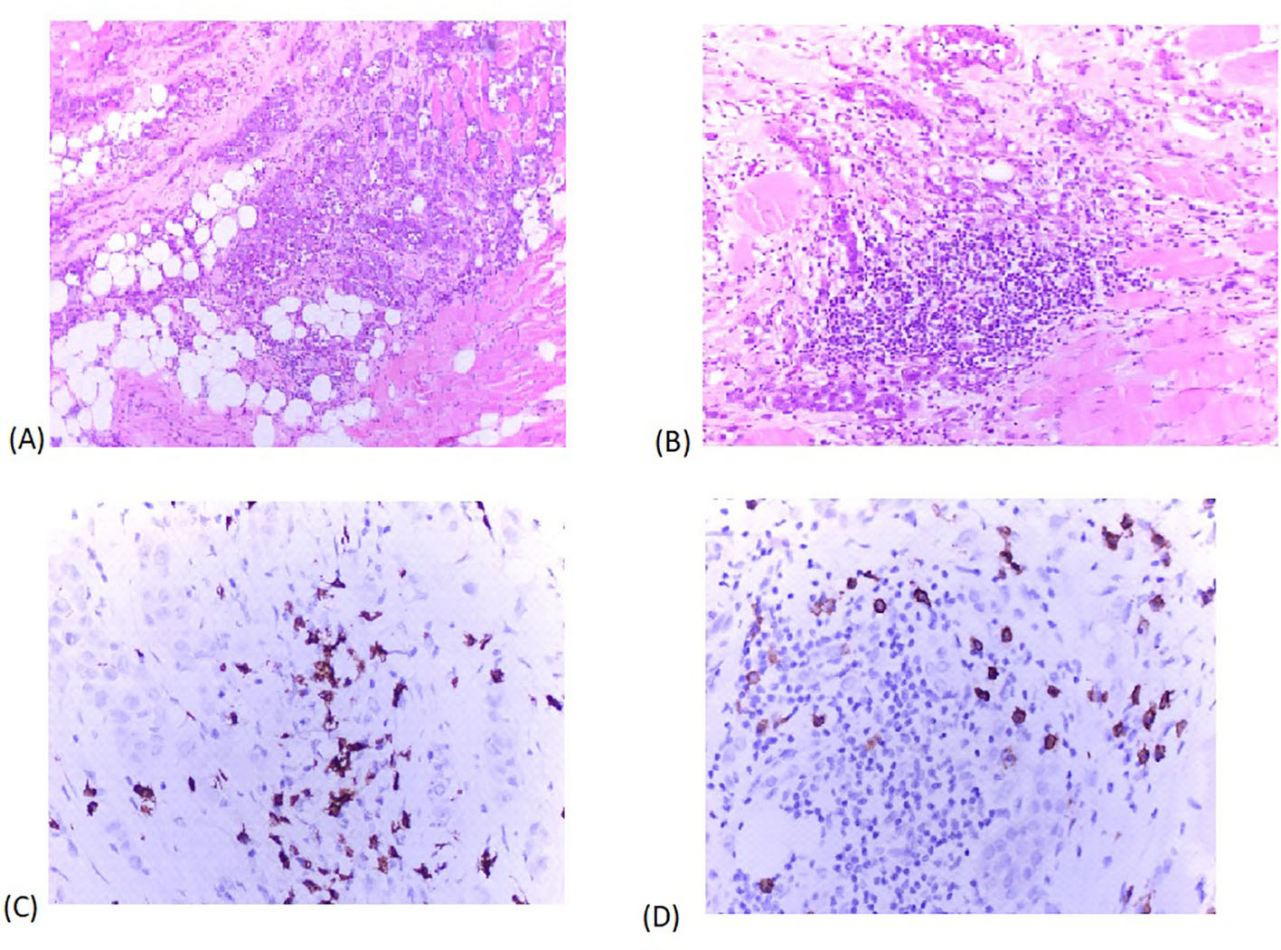
**(A)**. H&E x 200 tumor tissue is composed of infiltrative pseudoglandular structures lined by atypical mesothelial cells – embedded in desmoplastic stroma infiltrated with abundant mononuclear cell infiltrate. **(B)**. H&E x 400 small lymphoid aggregates in close proximity of tumor tissue. **(C)**. Immunohistochemical staining x 400 Inflammatory infiltrate is characterized with high proportion of cytotoxic, CD8+ lymphocytes. **(D)**. Immunohistochemical staining x 400 CD138 positive plasma cells in tumor immune microenvironment.

Microscopically, tumor tissue is composed of infiltrative pseudoglandular structures lined by atypical mesothelial cells – embedded in desmoplastic stroma infiltrated with abundant mononuclear cell infiltrate (**Figure 4A**) with occasional formation of small lymphoid aggregates (LA) (**Figure 4B**). Immunohistochemically, PD-L1 TPS (tumor proportion score) assessed with PD-L1 clone SP253, Ventana, was 2%. Inflammatory infiltrate is characterized with high proportion of cytotoxic, CD8+ lymphocytes (**Figure 4C**) and CD138 positive plasma cells (**Figure 4D**).

We performed FoundationOne^®^CDx analyses on primary tumor tissue of our patient in February 2022. in order to reveal possible gene alterations explaining the observed therapeutic efficacy of pembrolizumab. Analyses showed that this sample is microsatellite-stable (MSS), without mutations in microsatellite gene loci. Tumor Mutational Burden (TMB, also known as mutation load) in our patients’ tumor sample was 1 Muts/Mb. BAP1 (BRCA1 associated protein-1) gene alteration S438 and PBRM1 (Polybromo-1) gene deletion exon 19 were determined in our case.

## Discussion

In this report, we present a clinical course of a patient with advanced epithelioid type PM in whom standard chemotherapy treatment (ciplatin and pemetrexed combination) was started after surgery with postoperative residual disease. Despite early progression on first-line therapy, the patient experienced a long progression-free interval on second-line chemotherapy-bevacizumab combination, and finally a complete long-lasting response on third-line immunotherapy with pembrolizumab.

Polychemotherapy with cisplatin and pemetrexed is a standard treatment for patients with non-resectable PM. It was approved by the US FDA in February 2004., based on the pivotal study by Volgelzang et al. that showed that cisplatin in combination with pemetrexed achieved median OS of 12.1 months, which was significantly longer compared to patients who received only cisplatin (median OS of 9.3 months) (6). Unfortunately, our patient has rapid disease progression on first-line treatment.

The rationale for choosing second-line gemcitabine-bevacizumab (an anti-VEGF monoclonal antibody) treatment in this patient were findings of previous preclinical and clinical studies. Anti-VEGF blockage was shown to have a potential to suppress the progression of mesothelioma cells, and first-line combination therapy of bevacizumab with cisplatin and pemetrexed led to prolonged OS compared to the same chemotherapy alone (10, 13). Still, at this time, there are no second-line positive trials assessing this combination in patients with pleural mesothelioma. Most promising results came from RAMES study which explored another angiogenic drug ramucirumab, in combination with gemcitabine, and showed enhanced clinical activity of this approach (12). Second-line bevacizumab in combination with erlotinib was tested in a small open-label multicenter study that showed stable disease in 5 patients (21%) that lasted for at least 6 months, and 1 patient continued with this treatment for 21 months. The factors most closely associated with better outcomes in that trial were an ECOG performance status of 0, epithelioid histology, and a baseline white blood cell count that was not elevated (14). By clinical and tumor characteristics, our patient resembled patients with a good response in that study, and indeed achieved an extraordinary result on second-line treatment with gemcitabine-bevacizumab combination. The patient had disease control for 14 months, which largely surpasses expected progression-free survival (PFS) for second-line gemcitabine chemotherapy in PM (median 3 to 4 months) (1).

Immunotherapy with checkpoint inhibitors that has revolutionized treatment of many cancer types has also found place in the treatment of unresectable PM patients. Through immunotherapy, the immune system is activated to trigger an effective antitumor response. The inhibition of the PD-1, PD-1 ligand-1 (PD-L1), and CTLA-4 has been explored in a major way in PM treatment. Since only therapy in the third-line treatment of our patient was palliative in nature, pembrolizumab monotherapy was started with the patient’s consent.

The efficacy and safety of pembrolizumab in later lines of therapy have been tested in clinical studies in patients with advanced MPM. Furthermore, some good results were achieved in patients who did not show improvements with previous chemotherapy. Alley et al., in the KEYNOTE-028 Phase Ib trial, reported that a partial metabolic response to pembrolizumab was observed in 5 out of 25 patients (20%) with PD-L1 positive PM for whom standard therapy did not work. Furthermore, 13 patients (52%) achieved stable disease with a disease control rate (DCR) of 72% (15).

Contrary to the exceptional effectiveness of pembrolizumab in our patient, are the results of the PROMISE-meso, phase III trial, which investigated the efficacy of pembrolizumab versus institutional choice single agent chemotherapy (gemcitabine or vinorelbine) in relapsed PM patients with progression after/on previous platinum-based chemotherapy. In biologically unselected patients, although associated with an improved objective response rate (ORR), pembrolizumab did not improve PFS or OS over single agent chemotherapy (16). Another PD-1 inhibitor nivolumab was tested in second- line treatment versus placebo in patients progressing on first- line platinum based chemotherapy (CONFIRM trial). Immunotherapy with nivolumab led to significantly prolonged PFS [HR 0.67 (95% CI 0.3–0.85; p=0.0012)] and OS [median 10.2 months versus 6.9 months (adjusted HR 0.69 (95% CI 0.52–0.91); p=0.0090)] in comparison to placebo (17).

A systematic review on monotherapy with PD-1/PD-L1 checkpoint inhibitors in pretreated patients with advanced PM showed moderate antitumor activity of these drugs (response rate 18.1%, disease control rate 55.4%) with possibly higher activity in PD-L1 positive patients (18).

In 2020. US Food and Drug Administration (FDA) approved pembrolizumab for second-line treatment of advanced mesothelioma with high TMB based on the results of KN-158 (basket study). Nowdays, checkpoint inhibitors have found their place in first-line treatment of unresectable PM. The CheckMate 743 study evaluated nivolumab 3 mg/kg plus ipilimumab 1 mg/kg for up to 2 years compared to cisplatin or carboplatin plus pemetrexed for 6 cycles as first-line treatment in patients with unresectable PM. Experimental arm showed a 26% decrease in the risk of death in PM patients compared to standard chemotherapy (8). Most recently, the results of the IND 227 study have been reported: pembrolizumab/cisplatin/pemetrexed combination outperformed cisplatin/pemetrexed in terms of PFS and OS at the cost of somewhat higher toxicity (9). We lack long-term results from these studies and the true impact of unselectively applied immunotherapy in first-line PM treatment.

Additional pathohistological analysis in our patient revealed weak positive staining with PD-L1 tumor proportion score (2%). Some previous studies have shown a significantly worse prognosis in patients with PM positive for PD-L1 compared to those without PD-L1 (19). The median survival time was only 4.8-5 months in the former case, while for the latter it was 14.5-16.3 months (8, 19). Our patient (although the PD-L1 status was weakly positive and the TMB was low) had a complete response to pembrolizumab therapy and has been in clinical follow-up since September 2018.

On the other hand, this tumor is characterized by an unusual inflamed immune cell microenvironment, with lymphoid follicle formation, a high proportion of CD8+ cytotoxic tumor-infiltrating lymphocytes, and plasma cells, which suggests that the composition of the tumor immune microenvironment may influence response to immunotherapy (20). CD8+ lymphocytes are tumor suppressive and play a crucial role in the effectiveness of immune checkpoint blockade in cancer (21). Passelo detected that the CD + cell ratio increased after administration of pemetrexed and platinum (22). Recently, it was shown that long-term epitheloid PM survivors initially have higher intratumor presence of LA and B cells in comparison to short-term survivors (23). A review exploring the role of B-cell and plasma cell tumor infiltration in different solid tumor types pointed towards positive role of these cells in antitumor immunity, particularly when both of these cell types are present (24). The study by Patil et al. also suggests that plasma cells, which have long been considered to be a minor player in the development of antitumor responses, are strongly involved in the clinical responses to ICI in cancer patients. The underlying molecular and cellular mechanisms that make plasma cells an important predictor of clinical response to ICI treatment are unknown (25). Analogy of our case with other human malignancies highlight the importance of the coordination between cellular and humoral adaptive immune responses. Althogether, these findings can open the window of opportunity to identify fully human antibodies binding to unknown tumor associated antigens, as a novel immunotherapeutical approach (26).

Important findings came from work by Mangiante et al, who by using multiomics analyses made morphomolecular classification of PM based on four dimensions: ploidy, tumor cell morphology, adaptive immune response and CpG island methylator profile, that reflect tumor specialization (27). Tumors with pronounced markers of adaptive immune response histologically resemble our patients’ tumor sample, with lymphocyte infiltration and enrichment with B lymphocytes. By their analyses these tumors also often show high expression of hypoxia related genes, thus possibly explaining observed good therapeutical response achieved with bevacizumab and pembrolizumab in this patient. Previous results of French group also found that the continuum of different expression profiles in genes of immune response and angiogenesis describes better the behavior of PM than any discrete model (28).

Validation of the type of immune cell microenvironment, the role of the high ratio of CD8+ lymphocytes and plasma cells, the presence of LA, and its contribution to the therapeutic response in ICI in mesotheliomas requires further investigation.

Based on clinical evidence and the results of FoundationOne^®^ CDx analysis, MSS tumors are significantly less likely than MSI-H tumors to respond to anti-PD-1 immune checkpoint inhibitors, including approved therapies with nivolumab and pembrolizumab (29). Although in our case the patient’s tumor was MSS, a complete response to pembrolizumab has been achieved. Additionally, analyses in several solid tumor types reported that patients with higher TMB (defined as 16-20 Muts/Mb) achieved a greater clinical benefit from monotherapy with PD-1 or PD-L1 targeting drugs, compared to patients with higher TMB treated with chemotherapy or those with lower TMB treated with PD-1 or PD-L1 targeting agents (KEYNOTE 158) (30). Contrary to these analyses, our patient with TMB of only 1 Muts/Mb had a complete response to pembrolizumab. Subsequent analyzes of CM 743 also did not show a greater OS benefit of dual immunotherapy in patients with higher TBM (31). Therefore, the predictive value of TMB in PM is still questionable.

The inactivation of BAP1 is associated with prolonged survival in patients with mesothelioma (32). The BAP1 alterations such as S438* alteration that was found in our patient can lead to disruption of BAP1 function or expression (33). Loss of BAP1 protein expression predicted longer survival (16.1 months vs. 6.3 months) in patients with mesothelioma in one study, but was not prognostic in another (34, 35). Furthermore, patients with malignant mesothelioma and germline BAP1 mutations exhibited 7 times longer long-term survival compared to patients without germline BAP1 mutations (36).

Another gene alteration that was found in this patient’s tumor – PBRM1 inactivation – may predict the benefit of ICI targeting PD-1, such as nivolumab, pembrolizumab, cemiplimab, or dostarlimab, for patients with clear cell renal cell carcinoma and previous antiangiogenic therapy (37). However, multiple retrospective analyses report that the status of the PBRM1 mutation is not associated with the clinical benefit of various ICI in other solid tumor types, including non-small cell lung cancer, urothelial carcinoma, melanoma, or esophagogastric cancer, suggesting that the impact of loss of function of PBRM1 may depend on the type of tumor (38). There are no reports on its meaning in PM.

Obviously, there is a great need to conduct retrospective and prospective studies relating the histological findings of PM, especially on the tumor microenvironment, and the results of next generation sequencing with the results of immunotherapy treatment in these patients.

## Conclusions

Unfortunately, even today, pleural mesothelioma is usually detected late, in inoperable and advanced stages of the disease, leading to short survival and poor quality of life for these patients. This case review showed that some patients, although chemorefractory to the best chemotherapy option, can maintain great benefit of second-line chemotherapy in combination with antiangiogenic agent, and especially immunotherapy, even in late line of treatment. In this patient, immunotherapy with the PD-1 inhibitor pembrolizumab achieved a complete and prolonged response that transferred to long- term survival. Future research should focus on the identification of molecular, genetic, histological, and clinical biomarkers that could help us lead the selection of treatment individually for every patient in this rare disease.

## Data Availability

The original contributions presented in the study are included in the article/supplementary material, further inquiries can be directed to the corresponding author/s.
